# Patient's specific integration of OAR doses (D2 cc) from EBRT and 3D image‐guided brachytherapy for cervical cancer

**DOI:** 10.1002/acm2.12247

**Published:** 2018-01-19

**Authors:** Edgar Gelover, Cabel Katherine, Christopher Mart, Wenqing Sun, Yusung Kim

**Affiliations:** ^1^ Department of Radiation Oncology Mayo Clinic Rochester Minnesota USA; ^2^ College of Engineering Department of Biomedical Engineering The University of Iowa Iowa City IA USA; ^3^ Department of Radiation Oncology Medical University of South Carolina Charleston SC USA; ^4^ College of Engineering Department of Biomedical Engineering The University of Iowa Iowa City IA USA

**Keywords:** generalized equivalent uniform dose, HDR plan evaluation, high dose rate brachytherapy, OAR doses, overall D2 cc dose

## Abstract

The objective of this study was to assess the recommended DVH parameter (e.g., D2 cc) addition method used for combining EBRT and HDR plans, against a reference dataset generated from an EQD2‐based DVH addition method. A revised DVH parameter addition method using EBRT DVH parameters derived from each patient's plan was proposed and also compared with the reference dataset. Thirty‐one biopsy‐proven cervical cancer patients who received EBRT and HDR brachytherapy were retrospectively analyzed. A parametrial and/or paraaortic EBRT boost were clinically performed on 13 patients. Ten IMRT and 21 3DCRT plans were determined. Two different HDR techniques for each HDR plan were analyzed. Overall D2 cc and D0.1 cc OAR doses in EQD2 were statistically analyzed for three different DVH parameter addition methods: a currently recommended method, a proposed revised method, and a reference DVH addition method. The overall D2 cc_EQD__2_ values for all rectum, bladder, and sigmoid for a conformal, volume optimization HDR plan generated using the current DVH parameter addition method were significantly underestimated on average −5 to −8% when compared to the values obtained from the reference DVH addition technique (*P* < 0.01). The revised DVH parameter addition method did not present statistical differences with the reference technique (*P* > 0.099). When PM boosts were considered, there was an even greater average underestimation of −8~−10% for overall OAR doses of conformal HDR plans when using the current DVH parameter addition technique as compared to the revised DVH parameter addition. No statistically significant differences were found between the 3DCRT and IMRT techniques (*P* > 0.3148). It is recommended that the overall D2 cc EBRT doses are obtained from each patient's EBRT plan.

## 1 INTRODUCTION

Integration of concomitant chemotherapy, external beam radiotherapy (EBRT), and intracavitary brachytherapy (BT) is the standard of care in the curative management of locally advanced cervical cancer.^1^ Using a BT boost is linked with improved pelvic control^2^ and overall survival.^2^, ^3^ The first use of BT for the treatment of cervical cancer dates back to 1903.^4^ The use of three‐dimensional (3D) imaging techniques, such as computerized tomography (CT) and magnetic resonance imaging (MRI), have been rapidly replacing planar x‐ray imaging in BT treatment planning. This follows the recommendations of the Groupe Européen de Curiethérapie‐European Society for Therapeutic Radiology and Oncology (GEC‐ESTRO),^5^, ^6^, ^7^ the American Brachytherapy Society (ABS),^8^, ^9^ EMBRACE (An int**E**rnational study on **M**RI‐guided **BR**achytherapy in locally Advanced **CE**rvical cancer) protocol,^10^ and a recent International Commission on Radiation Units and Measurements (ICRU) Report #89.^11^ Volumetric dose parameters for targets and organs‐at‐risk (OARs) were introduced and used, allowing clinicians to customize isodose lines with the goal of achieving maximal coverage of the high‐risk clinical target volume (HR‐CTV) while irradiating OARs as little as possible. These adaptive, conformal BT approaches have resulted in significantly improved clinical outcomes.^12^ Volumetric OAR dose constraints, such as the minimal dose of the 2 cc of normal tissue with the highest dose (D2cc) or D0.1 cc, have been investigated^13^, ^14^, ^15^ as an alternative to conventional rectum and bladder point doses. These alternatives originated from the ICRU Report #38,^16^ and are mainly applicable to Point A‐based BT planning techniques. In order to integrate overall volumetric OAR doses (D2 cc and D0.1 cc) from EBRT and BT, it was recommended that the EBRT and BT doses be added even though the location of given hot‐spots (D2 cc or D0.1 cc regions) may not be identical for each of the plans. This was initially called a “worst case assumption” in the GEC‐ESTRO recommendation,^7^ but a worse case would occur due to intra‐fraction organ or applicator motion. The adopted EMBRACE protocol phrase for this is “DVH parameter addition”.^10^ In this DVH parameter addition technique, the EBRT component dose distributions (at least for the volumetric OAR parameters (D2 cc and D0.1 cc)), are assumed to be completely uniform EBRT prescription doses following the recommendations of the EMBRACE protocol.^5^, ^7^, ^10^

There have been efforts to accurately estimate overall doses from EBRT and HDR BT plans^17^, ^18^, ^19^ but he previous studies were performed using either a phantom study^17^ or a dosimetric planning study with no statistical analysis for six or fewer patients,^18^, ^19^ and they did not present a practical approach on how to estimate the overall OAR doses (e.g., D2 cc_EQD2_) without exporting and processing dose DICOM files (dose distribution (DVH_EQD2_) addition) or using DIR‐based DVH analysis. In this study, we present a practical revised DVH parameter addition method where the volumetric OAR parameters (e.g., D2 cc) are simply obtained from each patient's EBRT plan, instead of assuming a completely uniform EBRT prescription dose. The proposed, revised DVH parameter addition method was compared with the current DVH parameter addition method that has been used in the overall dose integration framework of GEC‐ESTRO guidelines^7^ and the EMBRACE protocol,^10^ and it assumes the completely uniform EBRT prescription dose. A dose distribution (DVH_EQD2_) addition was used as a reference dataset to compare those two approaches. Two different BT planning techniques (a) conventional Point A HDR plans and (b) 3 Tesla MRI‐guided, conformal, and adaptive volume optimization HDR plans were examined. In addition, the integrated, single EQD2‐based DVH, generalized equivalent uniform doses (gEUD_EQD2_) for the rectum, bladder, and sigmoid are presented and compared with conventional D2cc values as a potential, additional plan evaluation metric for OAR.

## 2 MATERIALS AND METHODS

### 2.A EBRT and 3T MRI‐guided, adaptive/conformal volume optimization HDR plans

Following the approval from our institutional review board (IRB), a retrospective study was performed with 31 biopsy‐proven cervical cancer patients whose International Federation of Gynecology and Obstetrics (FIGO) stages varied from Ib to IV. All patients received EBRT and HDR BT treatments. IMRT (10 patients) or 3D‐conformal EBRT (21 patients) plans were clinically generated in Pinnacle^3^ v9.8 (Philips Healthcare, Inc., Thornton, CO, USA). The type of EBRT planning techniques was clinically determined and the EBRT plans were retrospectively analyzed. All EBRT plans had HDR boosts in which an HDR plan was created for each fraction using BrachyVision v11.0 (Varian Medical System, Inc. Palo Alto, CA, USA). Patients were prescribed 45 Gy in 25 fractions, and 12 patients each received a 5.4–10 Gy EBRT boost in 3–5 fractions due to the involvement of PM (13 patients) or paraaortic (PA) (5 patients) regions. Two patients received both PM and PA EBRT boosts. Standard Point A‐based HDR plans for use with a Fletcher‐Suit‐Declos tandem‐and‐ovoids (T&O) applicator (Varian Medical System, Inc.) were clinically generated according to the ABS consensus guidelines.^8^, ^9^ The prescription dose was 33–36 Gy in 5–7 fractions, typically 5.5 Gy × 5 fractions or 7 Gy × 4 fractions). The clinical Point A plans were generated on 3 Tesla T2‐ and T1‐weighted MRI data sets^20^ (MAGNETOM Trio^TM^, Siemens Medical System Inc., Erlangen, Germany). A staff physician contoured the bladder, rectum, and sigmoid structures using T2‐weighted MR images.^11^ A T&O applicator was reconstructed (digitized) on T1‐weighted MR images.^6^ The details of the HDR workflow, imaging, and planning have been previously described.^21^, ^22^, ^23^, ^24^

**Figure 1:**
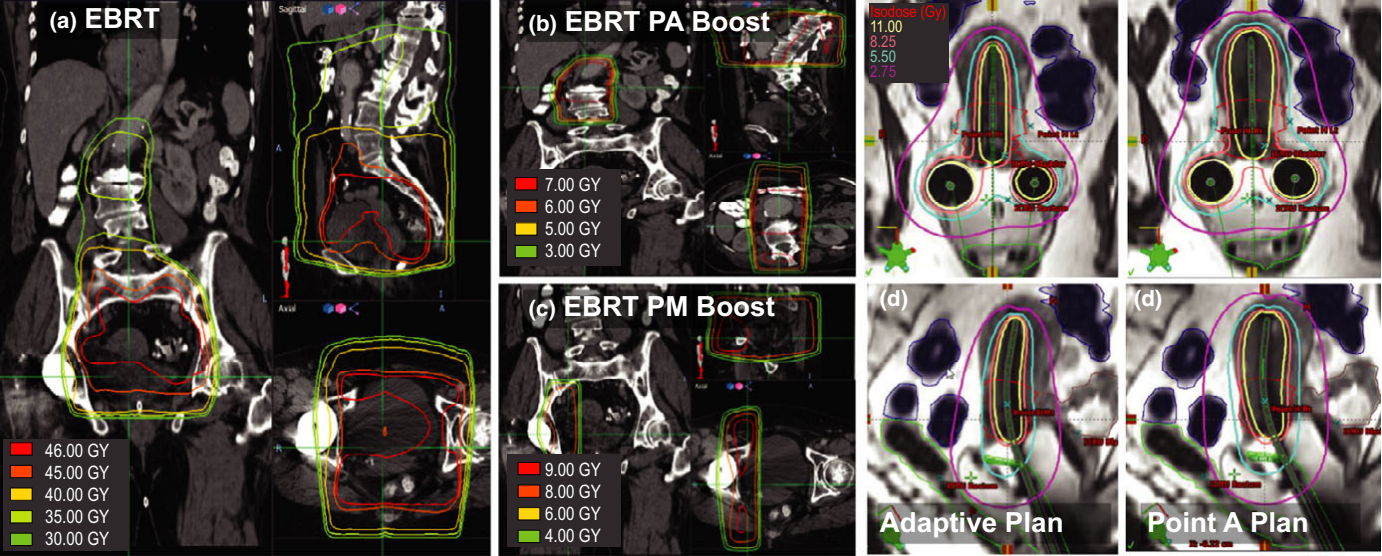
A case of EBRT (45 Gy) (a) with paraortic boost (PA: 7.2 Gy) (b) and parametria boost (PM: 9 Gy) (c) received with a Point A HDR plan (e) that retrospectively regenerated as a volume optimization, adaptive HDR plan (d).

An adaptive/conformal volume optimization HDR plan was retrospectively created for each clinical Point A plan through a hybrid‐inverse optimization process that includes a combination of an inverse optimization and manual forward planning. The hybrid‐inverse optimization process^21^, ^22^, ^25^, ^26^ includes three main steps: (a) generate a conventional Point A plan, (b) set dose‐volume objective constraints for inverse optimization based upon the resulting DVH parameters, and (c) perform final dose shaping using graphical optimization based upon DVH parameters and isodose lines. As a last step, a physician reviews the isodose lines for each slice on coronal, sagittal, and axial views. A patient's initial EBRT plan isodose lines, PA boost, and PM boost are depicted in Fig. 1, along with two different HDR plan techniques: a Point A plan and conformal volume‐optimization plan.

### 2.B Two different techniques to access overall OAR DVH parameters (D2 cc and D0.1 cc)

Two different approaches to assess overall OAR DVH parameters (e.g., D2 cc and D0.1 cc) from EBRT and each HDR plan were tested. The first is the recommended GEC‐ESTRO^7^ and EMBRACE protocol^10^ DVH parameter addition technique where a completely uniform EBRT prescription dose is assumed. EBRT DVH parameters (e.g., D2 cc) were assumed to receive the full EBRT prescription dose from the initial EBRT plan and PA boost but receive no additional doses from the PM boost due to its central block. A 4 cm central block was used for all EBRT PM boost plans. The second method is a revised DVH parameter addition technique where EBRT DVH parameters (e.g., D2 cc) are obtained from each patient's EBRT plan. In both approaches, their physical D2 cc and D0.1 cc parameters were converted into EQD2‐based values (D2cc_EQD2_ and D0.1cc_EQD2_) according to a linear‐quadratic cell survival model following GEC‐ESTRO guidelines^5^, ^7^ and the ICRU Report #89.^11^ The α/β ratio of 3 and repair halftime $\left(T_{1/2}\right)$ of 1.5 hr were used.^11^

(1) $$EQD2=\frac{ND(1+g\frac{d}{\alpha /\beta })}{(1+\frac{2}{\alpha /\beta )}}$$

Here N, *d*, and *g* represent a fraction number, a dose per fraction, and an incomplete repair function that is 1 for HDR. Afterward, the DVH parameters (e.g., D2 cc) in EQD2 were added for each EBRT and HDR plan. Both approaches can be simply done using an Excel spreadsheet (Microsoft Corporation, Redmond, WA, USA) available as a template on the American Brachytherapy Society website (www.americanbrachytherapy.org).

### 2.C Integrated, single EQD2‐based DVH as a reference dataset

**Figure 2:**
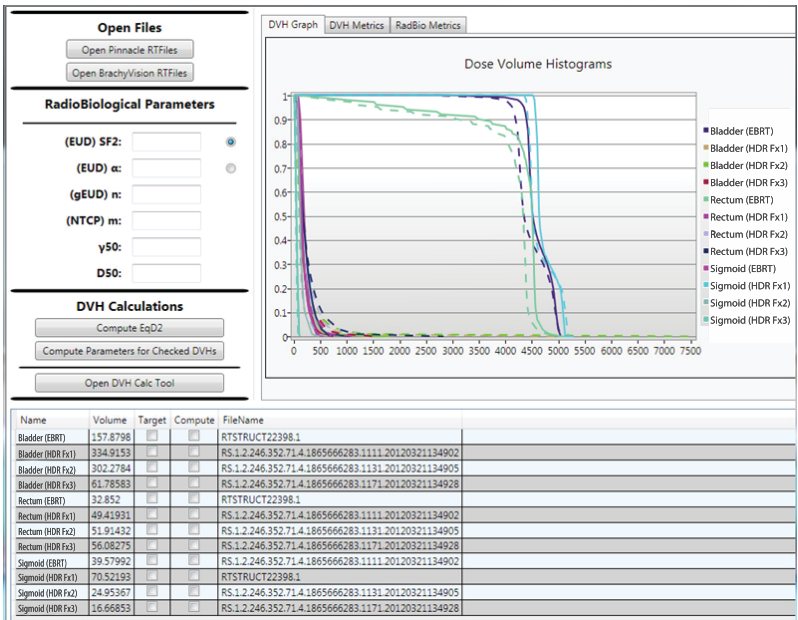
In‐house radiobiological evaluation tool (RadioBioEval) for integrated EBRT and each HDR brachytherapy plans through which physical EBRT and HDR DICOM dose map plans (solid lines on DVH Graph) are converted into EQD2‐dose maps and DVHs (dashed lines). In this demonstration case, a composite EBRT plan with EBRT boost and three HDR plans of fraction #1–#3 were imported.

In order to test these two different approaches, an integrated single EQD2‐based, differential DVH was generated as a reference dataset. This was done through three steps: (a) each physical dose map (i.e., dose DICOM file) was converted into an EQD2 dose map to account for the different fractionation schemes between the EBRT and HDR BT plans, (b) a differential DVH was generated from each EQD2 dose map that is EQD2‐based, differential DVH (DVH_EQD2_), and (c) all differential DVH_EQD2_ were combined to create a single, integrated differential DVH_EQD2_. The radiobiological plan evaluation tool, RadioBioEval, was developed in‐house as a stand‐alone software application in order to convert physical dose maps in DICOM format from EBRT treatment planning system (TPS) (Pinnacle, Philips Healthcare, Inc.,) and HDR TPS (BrachyVision, Varian Medical System, Inc.) into EQD2 dose maps, to generate a single, differential DVH_EQD2_ from EQD2 dose maps of EBRT and HDR plans and to evaluate an overall D2 cc_EQD2_, D0.1 cc_EQD2_, and gEUD_EQD2_ (see Fig. 2). A user put radiobiological parameters into “RadioBiological Parameters” of the RadioBioEval software to generate generalized EUD (gEUD) or other radiobiological metrics evaluated in the previous studies such as equivalent uniform dose (EUD) and tumor control probability (TCP)^27^ for tumor and normal tissue complication probability (NTCP).^28^ Afterward, DICOM dose files are imported through “Open Files” (seen in the left upper corner of the software, Fig. 2), then the imported file structures are presented in the bottom of Fig. 2. When “Target” is clicked, the α/β ratio of 10 is used. Otherwise, an α/β ratio of 3 is used. In this study, the α/β ratio of 3 was used for all OARs. In this demonstration case, a composite EBRT plan with EBRT boost and three HDR plans of fraction #1–#3 were imported. The DVH Graph of Fig. 2 presented the solid line DVHs of physical doses and the dashed line DVHs of EQD2 doses. When clicking “DVH Metrics” tab, the overall DVH parameters in EQD2 doses (e.g., D2 cc or D0.1 cc), referred to as the dose distribution addition (DVH_EQD2_), were obtained from the integrated, single, differential DVH_EQD2_. On “RadBio Metrics” tab in Fig. 2, gEUD and NTCP values for OARs and EUD and TCP values for tumors are presented.

### 2.D EQD2‐based, generalized EUD (gEUD_EQD2_) as an additional plan evaluation metric

Currently, the GEC‐ESTRO working group,^5^, ^7^ EMBRACE protocol,^10^ and the ICRU report #89^11^ all recommend assuming maximal dose–volume parameters. D2cc values sufficiently represent each OAR's dose distribution including whole DVH. As an additional plan evaluation metric, the use of an EQD2‐based, generalized equivalent uniform dose (gEUD_EQD2_) was proposed. The gEUD_EQD2_ was obtained from the integrated, differential DVH_EQD2_ that was described in the previous section. The gEUD_EQD2_ is determined by solving the following eq. (2):

(2) $$gEUD_{EQD2}={\left[\sum_{i}v_{i}\cdot \mathrm{EQD}2_{i}^{\frac{1}{n}}\right]}^{n}$$

where *n* is a volume effect parameter, and *EQD2*_*i*_ is the differential dose bin obtained from a single integrated, differential DVH_EQD2_. The *n* values of the rectum, bladder, and sigmoid were 0.23,^29^ 0.5,^30^ and 0.17,^30^ respectively, and were based upon the available literature. The values of gEUD_EQD2_ were compared with the values of current OAR plan evaluation metric, D2cc_EQD2_.

### 2.E Statistical correlation analysis

The percent differences of the two different DVH parameter addition techniques were statistically analyzed in comparison with the reference dataset. The statistical differences between the two were also measured. The impact of EBRT techniques such as 3D conformal radiotherapy (3DCRT) and intensity‐modulated radiotherapy (IMRT) were examined for the EBRT cases without PA or PM boosts. The consequence of PM boost contributions were evaluated by comparing a DVH parameter addition with no PM boost assumption method and the revised DVH parameter addition method. All analysis was separately performed for Point A and conformal volume optimization HDR plans, along with separate statistical analysis on each rectum, bladder, and sigmoid. All *P* values were calculated based upon paired, two‐sample Student t‐tests.

## 3 RESULTS AND DISCUSSION

The overall D2cc_EQD2_ parameters for the rectum, bladder, and sigmoid that were obtained from a revised DVH parameter addition technique presented no statistical differences (*P* > 0.0981) with the reference dataset values regardless of conformal, volume optimization, and Point A HDR plans (see Table 1). The overall D0.1cc_EQD2_ parameters for the rectum and sigmoid also did not present statistically significant differences between the revised DVH parameter addition and the reference dataset (*P* > 0.3831). Only the overall D0.1cc_EQD2_ values of the bladder were found to be significantly higher (*P* < 0.001) in the revised DVH parameter addition for either Point A or conformal HDR plan with a 5.9%–7.2% (7.5–7.8 Gy_EQD2_) average difference from the reference datasets. It is recommended that D0.1cc_EQD2_ values be recorded and monitored but with no dose limits.^5^, ^7^, ^10^, ^11^ When the overall D2cc_EQD2_ and D0.1cc_EQD2_ values were obtained from the current DVH parameter addition technique, all OAR values were significantly underestimated over the values of the reference dataset, regardless of HDR planning techniques (*P* < 0.01) (see Table 1). On average significantly lower D2cc_EQD2_ for all OAR were recorded than the values of the reference dataset in both conformal HDR plans (−4.7 to −8.3% (−2.8 to −5.6 Gy_EQD2_)) and Point A plans (−2.5 to −5.7% (−1.7 to −3.8 Gy_EQD2_)). The overall D2cc_EQD2_ values were underestimated over the reference dataset for the rectum, bladder, and sigmoid. The values reached as low as −11.7% (−6.9 Gy_EQD2_), −18.5% (−13.1 Gy_EQD2_), and −18.2% (−9.2 Gy_EQD2_), respectively. The overall D2cc_EQD2_ values were also significantly underestimated over the revised DVH addition technique (*P* < 0.004) for conformal and Point A HDR plans, reaching −4.2 to −6.0% (−2.8 to −4.6 Gy_EQD2_) and −4.1 to −5.8% (−3.1 to −4.8 Gy_EQD2_), respectively (see Table 2). The current DVH parameter addition technique resulted in significantly underestimated DVH parameter values (D2cc_EQD2_ and D0.1cc_EQD2_) for all OARs regardless of Point A or conformal HDR planning technique when compared to either the revised DVH parameter addition or the dose distribution (DVH_EQD2_) addition technique.

**Table 1:** The differences of DVH parameters (i.e., D2 cc and D0.1 cc) between either current DVH parameter addition or the proposed, revised DVH parameter addition and the reference dataset (i.e., either current DVH parameter addition or the revised addition — the reference dataset). The rectum, bladder, and sigmoid D2 cc_EQD2_ values of the revised DVH parameter addition did not present significant difference with the reference dataset (*P *>* *0.097) regardless of HDR planning techniques; for both conformal volume optimization and Point A plans

	∆ D2cc_EQD2_ [% (Gy_EQD2_)]		*P*‐value	∆ D0.1 cc_EQD2_ [% (Gy_EQD2_)]		*P*‐value
	Mean*	Std Dev		Mean*	Std Dev	
Current DVH parameter addition with Uniform EBRT Rx Dose Assumption						
Conformal HDR Plan						
Rectum	−4.7 (−2.8)	4.8 (3.1)	<0.001	−5.0 (−3.4)	5.1 (4.0)	<0.001
Bladder	−7.4 (−5.6)	5.9 (4.2)	<0.001	−13.0 (−12.9)	8.9 (8.5)	<0.001
Sigmoid	−8.3 (−4.9)	4.9 (2.6)	<0.001	−7.9 (−5.4)	5.2 (3.3)	<0.001
Point A HDR Plan						
Rectum	−2.5 (−1.7)	5.2 (3.4)	0.009	−4.0 (−3.3)	7.3 (6.3)	0.007
Bladder	−4.1 (−3.2)	6.0 (5.4)	0.002	−11.3 (−12.8)	6.1 (8.3)	<0.001
Sigmoid	−5.7 (−3.8)	6.2 (4.0)	<0.001	−5.8 (−5.1)	7.2 (7.3)	<0.001
Revised DVH parameter addition						
Conformal HDR Plan						
Rectum	−0.1 (+0.02)	5.6 (3.4)	0.980	+0.2 (+0.3)	5.2 (3.8)	0.720
Bladder	−1.2 (−1.0)	6.2 (4.9)	0.332	−7.2 (−7.8)	9.1 (9.3)	<0.001
Sigmoid	−1.6 (−0.8)	6.6 (3.8)	0.297	−1.4 (−0.8)	6.3 (4.4)	0.383
Point A HDR Plan						
Rectum	+1.8 (+1.4)	6.9 (4.6)	0.098	+0.7 (+0.6)	7.9 (7.0)	0.621
Bladder	+1.5 (+1.6)	6.4 (6.0)	0.152	−5.9 (−7.5)	6.9 (8.9)	<0.001
Sigmoid	+0.5 (+0.5)	7.1 (5.0)	0.551	+0.2 (−0.2)	7.5 (7.7)	0.872

Negative sign refers the values of current/revised DVH parameter addition underestimate when compared to the reference datasets.

**Table 2:** The differences of DVH parameters (i.e., D2 cc and D0.1 cc) when current DVH parameter addition and the proposed, revised DVH parameter addition were directly compared (i.e., current DVH parameter addition — the revised addition). The rectum, bladder, and sigmoid D2 cc_EQD2_ and D0.1 cc_EQD2_ values of the current DVH parameter addition were statistically significantly underestimated when compared to the revised DVH parameter addition (*P* < 0.0027) regardless of HDR planning techniques; for both conformal volume optimization and Point A plans

	∆ D2 cc_EQD2_ [% (Gy_EQD2_)]		*P*‐value	∆ D0.1 cc_EQD2_ [% (Gy_EQD2_)]		*P*‐value
	Mean*	Std Dev		Mean*	Std Dev	
Conformal HDR Plan						
Rectum	−4.2 (−2.8)	6.5 (4.1)	0.003	−4.7 (−3.7)	6.0 (4.3)	<0.001
Bladder	−5.6 (−4.6)	5.4 (4.5)	<0.001	−5.1 (−5.1)	4.5 (4.5)	<0.001
Sigmoid	−6.0 (−4.0)	7.2 (4.2)	<0.001	−6.0 (−4.6)	6.2 (4.1)	<0.001
Point A HDR Plan						
Rectum	−4.1 (−3.1)	5.5 (3.7)	<0.001	−4.4 (−3.9)	5.3 (4.0)	<0.001
Bladder	−5.3 (−4.8)	5.1 (4.3)	<0.001	−4.8 (−5.3)	4.4 (4.3)	<0.001
Sigmoid	−5.8 (−4.4)	6.2 (4.0)	<0.001	−5.6 (−4.9)	5.2 (4.0)	<0.001

Negative sign refers the values of current DVH parameter addition underestimate when compared to the revised DVH parameter addition.

No statistically significant differences in D2cc_EQD2_ values were recorded between two different EBRT techniques: 3D conformal radiotherapy (3DCRT) and intensity‐modulated radiotherapy (IMRT) cases for both conformal and Point A HDR plans, regardless of the rectum, bladder, or sigmoid (*P* > 0.3148) (see Table 3). For all PM boost cases, the current DVH parameter addition method assumes no contributions from 4 cm central block PM boosts. When directly compared to the revised DVH parameter addition method using values obtained from each patient's EBRT plan, D2 cc_EQD2_ and D0.1cc_EQD2_ values for all rectum, bladder, and sigmoid, regardless of conformal or Point A HDR planning technique, were statistically significantly underestimated (*P* < 0.008) (Table 4). On average, −8 to −10% (−5.4 to −8.0 Gy_EQD2_) underestimation occurred overall for OAR doses for the conformal HDR plans.

**Table 3:** The differences of D2 cc values between the reference dataset and either original DVH parameter addition or a revised addition method when two different EBRT techniques, 3DCRT and IMRT were compared. EBRT cases with PA or PM boosts were excluded for this analysis. No statistically significant differences were recorded (p > 0.3148)

Organ	3DCRT [% (Gy_EQD2_)]^a^		IMRT [% (Gy_EQD2_)]^a^		Difference Between 3DCRT and IMRT [% (Gy_EQD2_)]^b^	
	∆D2cc_EQD2_ Mean	∆D2cc_EQD2_ Std Dev	∆D2cc_EQD2_ Mean	∆D2cc_EQD2_ Std Dev	∆D2cc_EQD2_ Mean	*P*‐value
Current DVH parameter addition with Uniform EBRT Rx Dose Assumption						
Conformal HDR Plan						
Rectum	−3.8 (−2.3)	1.8 (1.2)	−3.9 (−1.9)	8.4 (5.3)	0.1 (−0.4)	0.9053
Bladder	−5.0 (−3.7)	9.2 (6.3)	−6.2 (−4.8)	2.9 (2.3)	1.2 (1.1)	0.6913
Sigmoid	−8.2 (−4.7)	5.4 (2.8)	−7.1 (−4.5)	2.5 (1.5)	−1.1 (−0.2)	0.4407
Point A HDR Plan						
Rectum	−2.1 (−1.4)	3.1 (2.2)	−3.6 (−2.3)	6.7 (4.6)	1.5 (0.9)	0.8845
Bladder	−3.9 (−3.1)	5.4 (4.9)	−1.8 (−1.0)	7.6 (7.7)	−2.1 (−2.1)	0.7509
Sigmoid	−7.3 (−4.7)	5.9 (2.7)	−4.9 (−3.5)	6.8 (5.0)	−2.4 (−1.2)	0.8157
Revised DVH parameter addition						
Conformal HDR Plan						
Rectum	−3.4 (−2.0)	8.6 (4.9)	−1.9 (−1.1)	1.2 (0.7)	−1.5 (−0.9)	0.9949
Bladder	−2.2 (−1.9)	10.0 (7.6)	−2.7 (−2.1)	1.6 (1.4)	0.5 (0.3)	0.5071
Sigmoid	−5.7 (−3.2)	9.4 (4.5)	−2.6 (−1.8)	1.8 (1.3)	−3.1 (−1.4)	0.3149
Point A HDR Plan						
Rectum	+1.6 (+1.4)	4.6 (3.5)	−1.3 (−0.8)	1.4 (1.0)	2.9 (2.2)	0.7352
Bladder	+0.5 (+0.6)	5.0 (5.0)	+1.2 (+1.8)	7.2 (7.8)	−0.8 (−1.2)	0.8419
Sigmoid	−0.4 (−0.4)	3.2 (2.4)	−0.7 (−0.5)	6.7 (5.5)	0.3 (0.1)	0.9156

a Negative sign refers the values of current/revised DVH parameter addition underestimate when compared to the reference datasets.

b The values = 3DCRT – IMRT. Thus, negative signs refers IMRT values are bigger than 3DCRT values.

**Table 4:** The differences of DVH parameters (i.e., D2cc and D0.1cc) only for PM boost cases between a DVH parameter addition with the assumption of no contributions from PM boost and a revised DVH parameter addition in which D2cc and D0.1cc parameters were obtained from each patient's EBRT plan (i.e., the revised DVH parameter addition — the current DVH parameter addition). The rectum, bladder, and sigmoid D2cc_EQD2_ and D0.1cc_EQD2_ values of the current DVH parameter addition were statistically significantly underestimated when compared to the revised DVH parameter addition (p < 0.0008) regardless of HDR planning techniques; for both conformal volume‐optimization and Point A plans

	∆ D2cc_EQD2_ [% (Gy_EQD2_)]		*P*‐value	∆ D0.1cc_EQD2_ [% (Gy_EQD2_)]		*P*‐value
	Mean*	Std Dev		Mean*	Std Dev	
Conformal HDR Plan						
Rectum	−7.9 (−5.4)	4.2 (2.7)	0.0003	−8.5 (−6.8)	3.1 (2.2)	<0.0001
Bladder	−9.3 (−8.0)	3.0 (2.7)	<0.0001	−7.8 (−8.3)	3.0 (2.8)	<0.0001
Sigmoid	−9.9 (−6.9)	3.4 (2.5)	<0.0001	−9.4 (−7.4)	2.6 (2.2)	<0.0001
Point A HDR Plan						
Rectum	−5.2 (−3.9)	6.8 (4.4)	0.007	−5.7 (−5.0)	6.7 (5.1)	0.004
Bladder	−7.1 (−6.5)	7.1 (6.0)	0.002	−6.3 (−6.8)	6.3 (6.1)	0.002
Sigmoid	−6.7 (−5.4)	8.7 (5.7)	0.005	−6.5 (6.0)	7.3 (5.6)	0.002

* Negative sign refers the values of current DVH parameter addition underestimate when compared to the revised DVH parameter addition.

The gEUD_EQD2_ values were statistically different from the current evaluation dose–volume parameters of D2 cc_EQD2_ values for all rectum, bladder, and sigmoid, regardless of which HDR planning technique was used (conformal or Point A HDR plans (*P* < 0.0001)) (see Table 5). All D2cc_EQD2_ values for the conformal HDR plan cases were measured as statistically significantly lower than those of Point A HDR plan cases (*P* < 0.02). For the rectum and sigmoid, the conformal HDR cases presented statistically significantly lower values than the Point A cases (*P* < 0.0496) but not for the bladder (*P* = 0.06423). The absolute D2cc_EQD2_ values were significantly higher than gEUD_EQD2_ values (*P* < 0.0001), since D2cc_EQD2_ values are maximal doses while gEUD_EQD2_ values are radiobiological mean doses accounting for the volume effect characteristics of each organ.

**Table 5:** The EQD2‐based gEUD values (gEUD_EQD2_) of integrated EBRT and HDR are compared with overall D2cc for each volume optimization, adaptive/conformal HDR plans, and Point A HDR plans

	D2cc_EQD2_ [Gy_EQD2_]			gEUD_EQD2_ [Gy_EQD2_]			*P*‐value
	Mean	Std Dev	Max	Mean	Std Dev	Max	
Conformal HDR Plan							
Rectum	64.3	7.5	80.9	52.2	5.7	64.1	<0.0001
Bladder	82.9	9.2	95.3	53.5	5.3	67.5	<0.0001
Sigmoid	65.7	5.7	75.9	56.1	4.6	68.5	<0.0001
Point A HDR Plan							
Rectum	70.3	10.8	93.0	55.3	5.9	72.0	<0.0001
Bladder	90.2	12.2	115.1	56.0	4.3	64.8	<0.0001
Sigmoid	74.3	14.2	122.3	60.8	6.7	79.1	<0.0001

In this study, we found that the overall D2cc_EQD2_ values for all rectum, bladder, and sigmoid for either conformal, volume optimization or Point A HDR plan that were generated through the current DVH parameter addition method, were significantly underestimated, when compared to the values obtained from the reference technique (*P* < 0.01) and the proposed, revised DVH parameter method (*P* < 0.004). The proposed, revised DVH parameter addition method, where the EBRT DVH parameters (e.g., D2cc) were simply obtained from each patient's EBRT plan instead of assuming a completely uniform prescription dose, did not present statistical differences with the reference dataset values (*P* > 0.099). In order to avoid significant underestimation of overall D2cc_EQD2_ values in clinical practice, it is recommended to simply obtain each patient's EBRT D2cc values of the rectum, bladder, and sigmoid from his or her EBRT plan instead of assuming a completely uniform prescription dose (the current DVH parameter addition) or exporting and processing dose DICOM files for each HDR plan (dose distribution (DVH_EQD2_) addition), which requires additional software. To more accurately estimate overall OAR doses by accounting for their locations, deformable image registration (DIR) techniques have been investigated.^31^, ^32^, ^33^, ^34^, ^35^

Van de Kamer et al. first tested the current DVH parameter addition method and found that it still yielded a sufficient approximation without an additional EBRT parametrial (PM) or paraaortic (PA) boost when compared to a dose distribution addition method (i.e., adding the EBRT and BT DVHs). However, they found that the overall D90 HR‐CTV with an EBRT PM (PA) boost for HDR was underestimated by 9.9 ± 6.2% (2.8 ± 1.4%) when a current DVH parameter addition method was used.^19^ They concluded that a “dose distribution (i.e., DVH) addition” method should be considered when an EBRT boost is given. Tamaki et al. highlighted the dosimetric impact of PM boosts with 3–4 cm central block^17^ through a simulated phantom setup. They found the contributions from central block of PM boost plan were on average 9% (5%–6%) and 28%–32% (11%–16%), for the D2cc_EQD2_ values of the rectum and bladder, respectively, when a 3 cm (4 cm) central block was used.^17^ Fenkell et al.^18^ also reported that an EBRT PM boost caused D2 cc_EQD2_ dose to increase by more than 50% over the boost prescription dose in four out of six patients and that the central PM boost shield does not predictably protect the high dose regions (D2 cc) of OARs. They proposed the use of intracavitary plus interstitial applicator treatments to properly cover the parametrial region instead of using the EBRT PM boost technique. Andersen et al. reported DIR‐based DVH parameter addition for the bladder was possible in 42 out of 77 cases (54%) and found mean deviations of 1.5 ± 1.8% in D2 cc EQD2 bladder values. They observed D2cc_EQD2_ dose deviations greater than 5% occurred in only 2% of the subjects, and they concluded that the recommended “current DVH parameter addition” provides a good estimate for the overall bladder D2cc_EQD2_ values. The Andersen et al. study^31^ included pathological node EBRT boosts but did not include PM boosts due to the use of tandem‐and‐ring (T&R) + interstitial applicators. It is worth noting that Andersen et al. could only test the overall D2cc values of the bladder by using a surface mesh DIR technique,^32^ but due to the considerable uncertainties of the DIR technique could not test the rectum, sigmoid, and small bowel. ICRU report #89^11^ states that “currently no deformable registration program is capable of tracking the location and dose‐exposure history of relevant biological structures within the target volumes and OAR”. Using DIR techniques is challenging mainly due to three factors: (a) 3D images of BT include vaginal packing with an applicator *in situ* and are not presented in EBRT image datasets; DIR algorithms assume there are paired pixels on two image datasets, (b) the significant deformation of organs such as the uterus and the sigmoid between EBRT and BT, and (c) the technical difficulties of reconciling DIR between MRI datasets which are the optimal 3D image guidance technique for BT, and CT datasets which are the most popular primary images for EBRT.

ICRU report #89^11^ addresses the assumption of a completely uniform EBRT dose that may not be valid for IMRT or volumetric‐modulated arc therapy (VMAT), and it identifies that fact that a special analysis is required when an EBRT PA or PM boost is used. When an EBRT PM boost is used, the evaluation of overall doses is especially challenging,^11^ since the central shield PM boosts do not predictably protect the OARs (D2cc_EQD2_). However, ICRU report #89 could not present a practical DVH parameter addition approach without processing the dose DICOM files for each EBRT and HDR plan.

One way to eliminate the complexity of dose integration of EBRT and BT is using EBRT boosts as an alternative to BT boost. Pioneering studies have investigated the feasibility of using IMRT or intensity‐modulated proton therapy (IMPT) boosts.^36^ Georg et al. concluded that 3D image‐guided, volume optimization, conformal BT techniques need to be used, since both advanced IMRT and IMPT boosts seem to be inferior to advanced BT boosts. For patients unable to undergo BT, stereotactic‐body radiotherapy (SBRT) techniques have been explored with different fractionation schemes such as 20 Gy in five fractions (23 Gy_10_ in EQD2),^37^, ^38^ 14 Gy in two fractions (20 Gy_10_ in EQD2),^38^, ^39^ 25 Gy in five fractions (31 Gy_10_ in EQD2),^40^ 5 Gy in three fractions (31 Gy_10_ in EQD2),^41^ or 6 Gy in five fractions (40 Gy_10_ in EQD2).^42^ 3D conformal radiotherapy (3D‐CRT) or IMRT techniques^38^, ^39^, ^41^, ^43^ along with CyberKnife (Accuracy Inc., Sunnyvale, CA, USA)^37^, ^40^, ^42^ have also been tested. However, the majority of clinical SBRT results^37^, ^38^, ^40^, ^42^ are still in the early stage with only small number of patients (<20 patients) and short‐term follow‐up (<26 months).

As preliminary data as a precursor to a large‐scale clinical outcome analysis, a radiobiological volumetric dose parameter (gEUD_EQD2_) which accounts for full DVH, and each organ's volume effect characteristic, such as serial or parallel organ, are presented here and demonstrated as significantly different from the current dose–volume plan evaluation metric (D2 cc_EQD2_). Clinical outcome (acute and late side effect) correlation analysis of using D2cc_EQD2_ alone, gEUD_EQD2_ alone, or a combination of D2cc_EQD2_ and gEUD_EQD2_ for OARs will be followed. Kim et al.^28^ presented the finding that overall EBRT gEUD_EQD2_ and each HDR plan has considerably higher statistical correlation with predicted normal tissue complication probability (NTCP) than the current overall D2cc_EQD2_. Shaw et al.^44^ proposed driving gEUD_EQD2_ criteria, resulting in conformal, volume optimization HDR plans per GEC‐ESTRO recommendations.^5^, ^7^ Yao et al. explored the use of gEUD as an inverse optimization objective function.^45^ The D2cc_EQD2_ OAR values are used as OAR plan evaluation metrics based upon the recommendations of the GEC‐ESTRO working group,^5^, ^7^ the EMBRACE protocol,^10^ and ICRU report #89^11^ that assumes that D2cc_EQD2_ HDR values or pulse dose rate (PDR) BT plans represent the full dose distribution (DVH) of each OAR reasonably well. As cautioned by ICRU report #89,^11^ the overall DVH pattern of OARs can change considerably with EBRT PA or PM boost, especially when IMRT or VMAT are used. Similar OAR DVHs pattern changes have occurred with BT treatments following the introduction of hybrid intracavitary and interstitial applicators such as the Vienna^46^, ^47^ or Urecht applicator.^48^ It is for these aforementioned reasons that similar overall D2cc_EQD2_ values, but significantly different volumetric doses (DVHs) are possible. As Koom et al. described, reporting organ doses in terms of a single point, or a maximal small volume dose assessment is not appropriate considering an inhomogeneous dose distribution, even in the event of a steep gradient over the high‐risk clinical target volume (HR‐CTV) and OARs near the source.^15^ In this study, all physical HDR plan doses (D2cc and D0.1 cc) were calculated based upon the AAPM TG 43 formula^49^, ^50^, ^51^ without heterogeneity‐corrections. The uncertainties of TG 43 dose calculations have been validated through a model‐based dose calculation algorithm that accounts for tissue and applicator heterogeneity.^52^ The uncertainties of OAR D2 cc in HDR BT plans for cervical cancer have been reported as on average 1%~3% for plastic tandem‐and‐ring (T&R) applicators calculated using a Grid‐Based Boltzmann Solver (GBBS) algorithm (Acuros, Varian Medical System, Inc.)^53^ or by a collapsed‐cone convolution algorithm (ACE, Elekta Ltd., Stockholm, Sweden).^54^ This is also true of titanium T&O applicators.^26^

## 4 CONCLUSIONS

The overall D2 cc_EQD2_ values for all rectum, bladder, and sigmoid for conformal, volume optimization HDR plans generated using the current DVH parameter addition method, (which assumes a completely uniform prescription dose of EBRT), were significantly underestimated by on average −4.7%~−8.3%, when compared to the reference technique of dose distribution (DVH) addition values (*P* < 0.01). The revised DVH parameter addition method where the EBRT DVH parameters (e.g., D2 cc) were simply obtained from each patient's EBRT plan did not present statistical differences with the reference technique's values (*P* > 0.099). When PM boosts were used, there was an average −8%~−10% underestimation of overall OAR doses occurred for conformal HDR plans when using the current DVH parameter addition technique when compared to the revised DVH parameter addition. No significant differences between 3DCRT and IMRT techniques were found when using different approaches to estimate the overall OAR DVH parameters.

## CONFLICT OF INTEREST

The authors declare that there is no conflict of interest.
